# Association between the systemic immuno-inflammation index and hearing loss: result from NHANES 2009–2018

**DOI:** 10.3389/fneur.2024.1369492

**Published:** 2024-04-23

**Authors:** Tingfeng Zhou, Jiesheng Mao, Pei Zhu, Xinru Yu, Xiaokai Yang

**Affiliations:** ^1^Postgraduate Training Base Alliance of Wenzhou Medical University (Wenzhou People’s Hospital), Wenzhou, China; ^2^The First Affiliated Hospital of Wenzhou Medical University, Wenzhou, China

**Keywords:** systemic immune-inflammatory index, inflammation, NHANES, hearing loss, otolaryngology

## Abstract

**Background:**

A novel inflammatory marker that measures the degree of systemic immunoinflammation, the systemic immuno-inflammation index (SII) is frequently used to forecast a number of illnesses. According to earlier studies, inflammation may play a role in the pathophysiology of hearing loss (HL).

**Methods:**

A sample from the National Health and Nutrition Examination Survey (NHANES) covering the years 2009 to 2018 was used in the current cross-sectional survey. Subgroup analysis and weighted multiple linear regression models were used to examine the independent linear correlation between SII and HL. Fitted smoothed curve analyses were also conducted to show the non-linear relationship between the two variables.

**Results:**

Among the 8,535 participants, the mean age was 40.92 ± 18.6 years, with 49.01% being male. Notably, individuals with hearing loss demonstrated an SII of 530.00 ± 320.72, while those with normal hearing displayed an SII of 491.21 ± 265.15. The mean ± SD values of low-frequency, speech-frequency, and high-frequency Pure Tone Average (PTA) hearing thresholds were 10.33 ± 9.79, 12.20 ± 11.11, and 22.48 ± 19.49 dB, respectively. A positive dose–response relationship between higher SII and hearing thresholds was observed after adjusting for potential confounders. Furthermore, the interaction analysis did not reveal any significant impact on this positive correlation.

**Conclusion:**

The results of our investigation suggest that the Systemic Inflammatory Index may serve as a potential biomarker for the likelihood of hearing loss. However, additional research is required to further elucidate the nature of this association.

## 1 Introduction

All age groups are affected by hearing loss (HL), a common sensory condition in otolaryngology. This illness causes considerable physical and mental anguish, ranking as the third most frequent cause of a lifetime of disability globally (1). It also has a massive influence on people’s daily lives. HL affects more than 100 million people worldwide; during the next 5 years, this number is predicted to double (2). HL is associated with a variety of genetic and environmental risk factors, such as congenital conditions, age, trauma, exposure to high noise levels, ototoxic medications, hypertension, diabetes mellitus, etc. (3, 4). Discovering and exploring the risk factors associated with HL and searching for their biomarkers are of great importance to the disease.

The course of HL may be significantly influenced by inflammation, according to previous observational studies that have connected higher plasma levels of inflammatory markers such as CRP, IL-6, and TNF-α with this illness (5–7). Furthermore, Neutrophil-to-lymphocyte ratio (NLR) and platelet-to-lymphocyte ratio (PLR) were employed as inflammatory indicators in a prior retrospective investigation, which discovered that sudden sensorineural hearing loss (SSNHL) was associated with considerably more significant levels of NLR and PLR than in healthy controls (5). NLR and PLR have been proven to be an essential predictor of the severity of sudden sensorineural hearing loss (SSNHL) in several case–control investigations (8–10). Hence, it is crucial to elucidate the influence of systemic inflammatory conditions on hearing loss. This is vital for gaining a comprehensive understanding of the role of inflammation in hearing loss and its potential as a therapeutic approach to improve overall health. The Systemic immuno-inflammation Index (SII) is a novel inflammatory marker computed as platelet count × neutrophil/lymphocyte count (11). SII has been revealed to be a prognostic indicator in individuals with several kinds of cancers, especially cervical cancer (12), colon cancer (13), and bladder cancer (14). Additional research suggests a connection between SII and other illnesses, among them diabetes (15), Chronic kidney disease (16), and cardiovascular disease (17). NLR and SII levels were considerably higher in 80 SSNHL patients, according to a recent retrospective study (18). This discovery raises the possibility that there is an inflammatory, immunological response, which could be significant for the management of hearing loss. Research on the relationship between SII and hearing loss is still in its infancy. In light of this, we used the SII indicator as an exposure factor in an attempt to explore whether this inflammatory marker is associated with hearing loss and whether it could become a biomarker of hearing loss in the future.

This study aimed to investigate the potential role of the Systemic Inflammatory Index as a biomarker for hearing loss in the United States population, utilizing data from the National Health and Nutrition Examination Survey (NHANES). The research sought to identify the effects of SII on hearing conduction and provide recommendations for the prevention and treatment of HL.

## 2 Methods

### 2.1 Study population and research design

**Figure 1:**
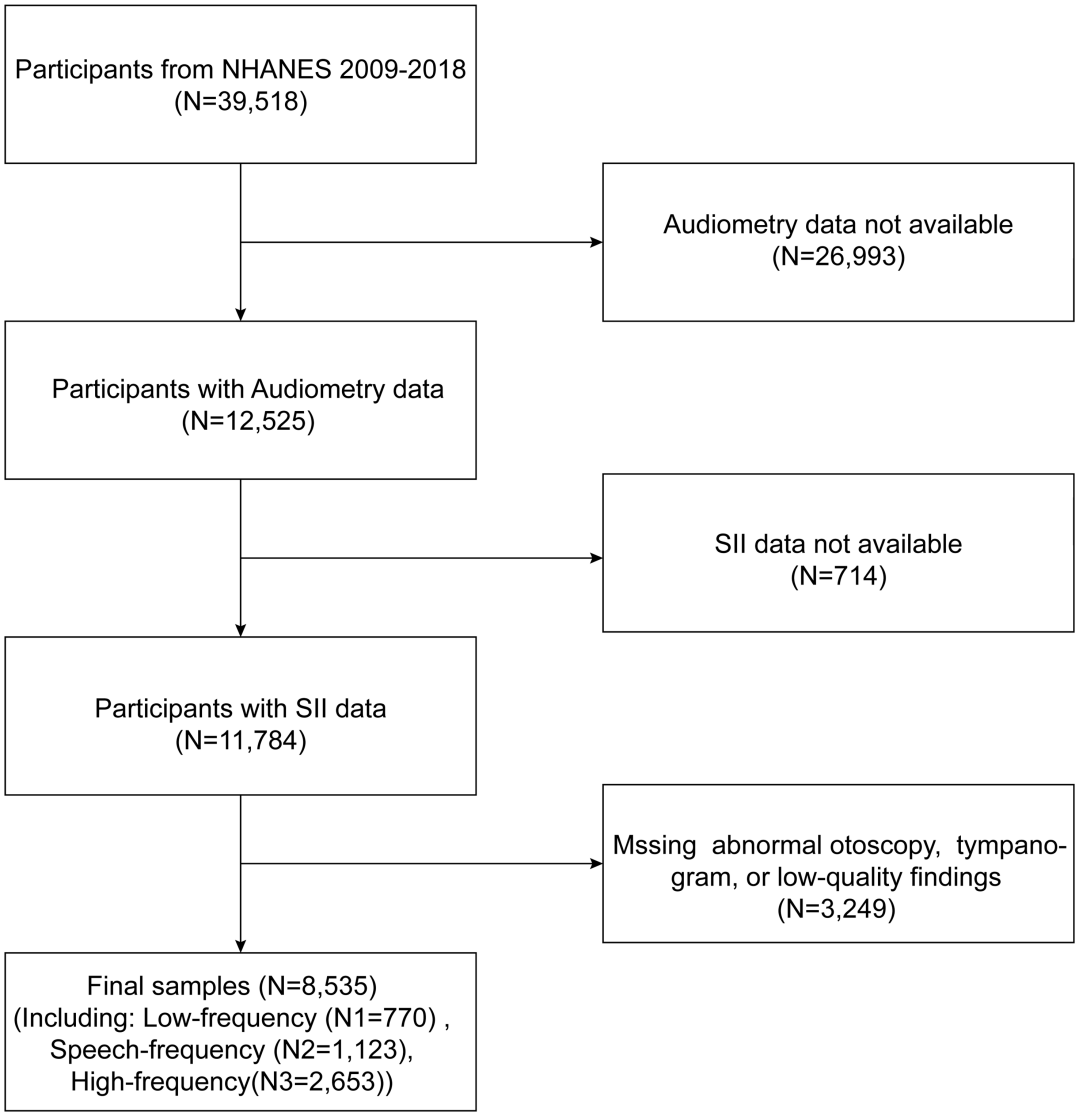
Flowchart of participant selection. NHANES, National Health and Nutrition Examination Survey.

Data from NHANES were used for the cross-sectional investigation, and data from 2009 to 2018 (apart from 2013 to 2014) were pooled to boost the sample size and improve the study’s reliability. The study offers unbiased data on the population of the United States’s nutritional status and risk factors. In total, e removed participants with incomplete audiometric data (*n* = 26,993) and incomplete SII data (*n* = 741). Additionally, patients with missing or abnormal otoscopy, missing tympanogram, or low-quality findings were omitted (*n* = 3,249) in order to reduce the impact of additional confounding factors. 8,535 qualified subjects in all were included in our analysis. Figure 1 shows a detailed flow chart explaining the selection procedure.

### 2.2 Audiometry

Pure-tone audiometry (PTA) was used as the outcome variable in this investigation. Participants performed normal air conduction PTA with an experienced and licensed audiologist in a soundproof environment. The participant’s hearing thresholds were assessed in both ears across the frequency range of 0.5–8 kHz. To guarantee the accuracy of the participant’s responses, each ear underwent two 1 kHz tests (19). The 0.5, 1, and 2 kHz hearing thresholds are averaged to determine the low-frequency (LF) hearing PTA. By averaging the hearing thresholds at 0.5, 1, 2, and 4 kHz, the speech-frequency (SF) hearing PTA is obtained. Finally, by averaging the hearing thresholds at 4, 6, and 8 kHz, the high-frequency hearing (HF) PTA is calculated. When one of the hearing thresholds was 25 dB or higher, it was considered to have a hearing loss.

### 2.3 SII and covariates

The exposure variable in this study was SII, which was based on a complete blood count. An automated hematology analyzer (CoulterDxH 800 Analyzer) was used to calculate the lymphocyte, neutrophil, and platelet counts, which were then expressed as 10^3^ cells/mL. We determined the SII by multiplying the platelet count by the neutrophil count/lymphocyte count, according to earlier studies (19, 20). Factors including age, gender (male or female), race (Mexican American, Non-Hispanic White, Non-Hispanic Black, Other Hispanic, and Other Race), education level (<high school, high school, and >high school), waist circumference, smoking status (whether you have smoked more than 100 cigarettes in your lifetime), alcohol consumption (such as consuming four or five drinks per day or more), diabetes, hypertension, and noise exposure were all taken into consideration in our study. Additionally, we incorporated the Oxidative Balance Score (OBS) as a covariate to assess oxidative stress. The OBS consists of 20 items, including dietary and lifestyle components. Each component represents different aspects of a healthy diet and lifestyle. Each component is scored according to specific criteria, as described in Supplementary Table S1.

### 2.4 Statistical analysis

Both EmpowerStats 2.0 and the R programming language (version 3.4.3) were used to do the statistical study. While continuous variables are typically described using measures such as mean plus or minus standard deviation (SD), categorical variables are often represented as percentages with weights. In this study, a weighted linear regression model was used to evaluate potential differences among individuals based on the SII tertile. Three models were developed to assess beta values and 95% confidence intervals for multiple linear regressions between SII and the audibility threshold. Variables were left unadjusted in Model 1, whereas in Model 2, adjustments were made for sex, age, and race. In contrast, Model 3 had adjustments for all covariates, including sex, age, race, education level, income-to-poverty ratio, BMI, waist circumference, noise exposure, alcohol consumption, hypertension, diabetes, and smoking status. Along with adjusting for these variables, smoothed curve fitting and subgroup analysis were carried out.

## 3 Results

### 3.1 Characteristics of participants

The study included 8,535 participants (weighted mean age 40.92 ± 18.66 years) recruited based on the inclusion/exclusion criteria and then stratified by SII tertiles. Remarkably, individuals with hearing loss demonstrated an SII of 530.00 ± 320.72, while those with normal hearing displayed an SII of 491.21 ± 265.15. This discrepancy indicates a significantly elevated SII among participants with hearing loss compared to those with normal hearing. Table 1 displays the clinical and biochemical characteristics of the subjects. Of the participants, 45.61% were male, and 54.39% were female. Among them, 2,975 (weighted, 34.85%) were classified as obese, 2,343 (weighted, 27.46%) had hypertension, and 690 (weighted, 8.09%) had diabetes. The mean ± SD values of low-frequency, speech-frequency, and high-frequency PTA hearing thresholds were 10.33 ± 9.79, 12.20 ± 11.11, and 22.48 ± 19.49 dB, respectively, and increased with increasing SII tertile. Of the total sample, 2,775 (weighted, 32.52%) participants experienced hearing loss, with a mean SII ± SD concentration of 503.83 ± 285.00. Additionally, there were differences in SII tertiles concerning age, waist circumference, platelet count, neutrophil count, Lymphocyte count, low-frequency, speech-frequency, and high-frequency PTA, BMI, race/ethnicity, income-to-poverty ratio, education, hypertension, diabetes, smoking status, and hearing loss rate. Table 2 presents the characteristics of participants based on the presence of LFHL, SFHL, and HFHL. In our study population, a total of 770 cases were classified as LFHL, 1123 cases were classified as SFHL, and 2,653 cases were classified as HFHL. These differences were statistically significant (all *p* < 0.05).

**Table 1:** Baseline characteristics of study participants in NHANES according to the tertiles of the systemic immune-inflammation index.

SII	Overall	Tertile 1 (≤349)	Tertile 2 (>349, ≤ 536)	Tertile 3 (>536)	*p* value
Continuous variables, mean ± SD					
Age (years)	40.92 ± 18.66	38.54 ± 19.25	41.11 ± 18.44	42.85 ± 18.10	<0.0001
BMI (kg/m^2^)	28.33 ± 7.17	26.81 ± 6.57	28.21 ± 6.84	29.82 ± 7.68	<0.0001
Waist circumference (cm)	96.45 ± 17.97	92.34 ± 17.46	96.56 ± 17.26	100.06 ± 18.32	<0.0001
Income to poverty ratio	2.97 ± 1.65	3.00 ± 1.66	2.98 ± 1.64	2.94 ± 1.66	0.3504
Platelet count (10^3^ cells/μL)	242.01 ± 58.63	211.02 ± 50.14	239.66 ± 47.80	271.93 ± 60.43	<0.0001
Neutrophil count (10^3^ cells/μL)	4.18 ± 1.65	2.94 ± 0.91	3.99 ± 1.04	5.48 ± 1.72	<0.0001
Lymphocyte count (10^3^ cells/μL)	2.17 ± 0.76	2.41 ± 0.92	2.18 ± 0.65	1.95 ± 0.63	<0.0001
Low-frequency PTA	10.33 ± 9.79	9.34 ± 9.27	10.36 ± 9.51	11.17 ± 10.41	<0.0001
Speech-frequency PTA	12.20 ± 11.11	11.07 ± 10.56	12.29 ± 10.90	13.11 ± 11.69	<0.0001
High-frequency PTA	22.48 ± 19.49	20.65 ± 18.76	22.92 ± 19.76	23.68 ± 19.72	<0.0001
Categorical variables, %					
Gender					<0.0001
Male	45.61	52.84	47.01	37.78	
Female	54.39	47.16	52.99	62.22	
Race					<0.0001
Mexican american	9.70	9.24	9.57	10.24	
Other hispanic	6.48	6.32	6.50	6.59	
Non-hispanic white	65.00	59.94	66.77	67.74	
Non-hispanic black	10.26	15.53	8.28	7.54	
Other races	8.57	8.97	8.89	7.90	
Education level					0.0041
Less than high school	13.21	14.19	11.62	13.93	
High school or GED	20.10	18.3	19.73	21.86	
Above high school	66.69	67.48	68.65	64.21	
Smoked at least 100 cigarettes	41.92	39.72	41.89	43.69	0.0213
Diabetes	8.09	6.69	8.08	9.34	0.0015
Had 4/5 or more drinks every day	15.34	14.78	14.93	16.18	0.3789
Hypertension	27.46	24.75	25.41	31.65	<0.0001
Noise exposure	29.29	28.99	28.22	30.56	0.3255
Hearing loss	32.52	28.92	33.41	34.85	<0.0001

**Table 2:** Baseline characteristics of study participants in NHANES according to hearing loss.

Characteristics	LFHL (*N* = 771)	*p* value	SFHL (*N* = 1,123)	*p* value	HFHL (*N* = 2,653)	*p* value
Continuous variables, mean ± SD						
Age (years)	65.90 ± 15.12	<0.0001	65.38 ± 14.13	<0.0001	527.45 ± 355.94	<0.0001
BMI (kg/m^2^)	29.27 ± 6.68	<0.0001	29.50 ± 6.53	<0.0001	29.78 ± 6.81	<0.0001
Waist circumference (cm)	101.48 ± 16.09	<0.0001	102.69 ± 15.57	<0.0001	102.41 ± 15.93	<0.0001
Income to poverty ratio	2.42 ± 1.52	0.722	2.44 ± 1.53	0.477	2.49 ± 1.60	0.043
SII	551.39 ± 357.27	<0.0001	565.86 ± 436.62	<0.0001	527.45 ± 355.94	<0.0001
Categorical variables,%						
Gender		<0.0001		<0.0001		<0.0001
Male	54.22		52.63		52.13	
Female	45.78		47.37		47.87	
Race		<0.0001		<0.0001		<0.0001
Mexican american	14.01		13.89		13.68	
Other hispanic	10.12		10.24		11.04	
Non-hispanic white	50.71		50.67		44.33	
Non-hispanic black	14.79		14.34		18.13	
Other races	10.38		10.86		12.82	
Education level		<0.0001		<0.0001		<0.0001
Less than high school	32.28		32.37		27.48	
High school or GED	23.02		24.30		23.86	
Above high school	44.71		43.34		48.67	
Smoked at least 100 cigarettes	48.68	<0.0001	52.26	<0.0001	49.85	<0.0001
Diabetes	24.90	<0.0001	25.82	<0.0001	21.18	<0.0001
Had 4/5 or more drinks every day	19.86	<0.0001	21.51	<0.0001	19.72	<0.0001
Hypertension	57.42	<0.0001	56.63	<0.0001	51.71	<0.0001
Noise exposure	33.27	<0.0001	37.02	<0.0001	36.71	<0.0001

The highest SII tertile group was more likely to be female and elderly than the lowest SII tertile group, and it had higher rates of smoking, alcohol use, diabetes, hypertension, exposure to noise, hearing loss, and measurements of BMI, waist circumference, platelet count, neutrophil count, LF PTA, SF PTA, and HF PTA.

### 3.2 The association between SII and hearing loss

Multivariate regression analyses were conducted to investigate the connection between SII and changes in PTA hearing thresholds for low, speech, and high frequencies. The outcomes are shown in Table 3. SII/100 was used to multiply the effect values by a factor of 100 in order to make the non-significant effect values more visible. When SII was viewed as a continuous variable in model 1, SII strongly connected with PTA hearing thresholds at low, speech, and high frequencies. After adjusting for all confounders, Model 2 and Model 3 both maintained the positive connection of SII with low-frequency, speech-frequency, and high-frequency PTA scores. For persons in the highest tertile of SII, every unit increase in SII was linked to a statistically significant increase in low-frequency (β = 0.86, 95%CI: 0.06–1.67; *p* = 0.0355) and speech-frequency (β = 0.91, 95%CI:0.08–1.73; *p* = 0.0308) PTA of 86 and 91%, respectively, in the fully adjusted model using the lowest tertile of SII as the reference value. Low-frequency and speech-frequency PTA did, however, also show a rising trend in the second tertile of SII, albeit this trend was not statistically significant. Furthermore, sensitivity tests for the third quartile of SII showed that participants’ high-frequency hearing threshold PTA increased along with the third quartile of SII, but no statistically significant association was found.

**Table 3:** Relationship between systemic immune inflammatory indices and hearing loss.

	β (95% CI), *p* value of PTA levels, dB		
	Model 1	Model 2	Model 3
Low-frequency PTA			
Continuous	0.28 (0.21,0.35) < 0.0001	0.12 (0.05,0.18) 0.0002	0.12 (0.01,0.23) 0.0288
Categories			
Tertile 1	Reference	Reference	Reference
Tertile 2	1.03 (0.51,1.54) < 0.0001	0.31 (−0.12,0.75) 0.1543	0.41 (−0.39,1.22) 0.3172
Tertile 3	1.84 (1.32,2.35) < 0.0001	0.69 (0.25,1.12) 0.0021	0.86 (0.06,1.67) 0.0355
*p* for trend	0.41 (0.29,0.52) < 0.0001	0.15 (0.06,0.25) 0.0021	0.19 (0.01,0.37) 0.0349
Speech-frequency PTA			
Continuous	0.32 (0.24,0.40) < 0.0001	0.13 (0.07,0.20) < 0.0001	0.14 (0.03,0.25) 0.0140
Categories			
Tertile 1	Reference	Reference	Reference
Tertile 2	1.22 (0.64,1.80) < 0.0001	0.34 (−0.11,0.79) 0.1385	0.28 (−0.54,1.11) 0.5049
Tertile 3	2.04 (1.46,2.62) < 0.0001	0.75 (0.29,1.20) 0.0012	0.91 (0.08,1.73) 0.0308
*p* for trend	0.45 (0.32,0.58) < 0.0001	0.17 (0.07,0.27) 0.0012	0.21 (0.03,0.40) 0.0231
High-frequency PTA			
Continuous	0.51 (0.37,0.66) < 0.0001	0.14 (0.04,0.24) 0.0048	0.18 (0.004,0.36) 0.0449
Categories			
Tertile 1	Reference	Reference	Reference
Tertile 2	2.26 (1.24,3.29) < 0.0001	0.45 (−0.25,1.14) 0.2059	−0.35 (−1.67,0.97) 0.6006
Tertile 3	3.03 (2.00,4.05) < 0.0001	0.50 (−0.20,1.20) 0.1597	0.59 (−0.73,1.91) 0.3800
*p* for trend	0.65 (0.41,0.88) < 0.0001	0.10 (−0.05,0.26) 0.1996	0.17 (−0.12,0.47) 0.2490

Model 1: no covariates were adjusted. Model 2: age, gender, and race were adjusted. Model 3: sex, age, race, education level, income-to-poverty ratio, BMI, waist circumference, noise exposure, alcohol consumption, hypertension, diabetes, smoking status, and OBS.

### 3.3 Subgroup analysis

**Figure 2:**
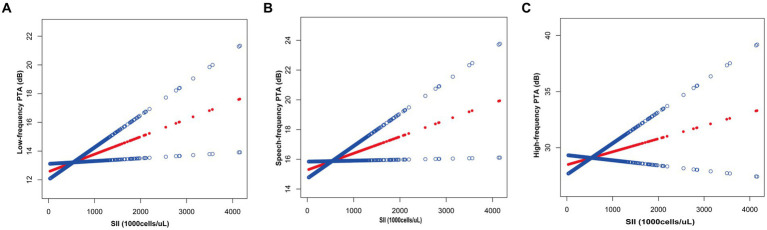
Relationship between SII and auditory threshold transfers: **(A)** Low-frequency PTA; **(B)** Speech-frequency PTA; **(C)** High-frequency PTA.

Based on Table 4, the subgroup analyses indicate that the correlation between SII levels and increased low-frequency PTA was not consistently observed. However, upon conducting subgroup analyses stratified by age, BMI, smoking status, hypertension, and diabetes, we found a significant association between SII and low-frequency PTA in subgroups of patients aged below 65 years, with a BMI below 30, smokers, and non-diabetics (all *p* < 0.05). The results of interaction tests indicated that there was no significant variability in the association between SII and low-frequency PTA across strata, indicating that the positive correlation was not significantly influenced by factors such as gender, age, BMI, diabetes mellitus, hypertension, smoking status, or alcohol consumption (*p* > 0.05 for all interactions). We performed a smoothed curve fit after correcting for relevant variables and discovered a correlation between SII and low-frequency, speech, and high-frequency PTA (Figure 2).

**Table 4:** The association between SII and LF PTA by selected subgroups.

Subgroup	Low-frequency PTA	
	β (95% CI), *p* value of PTA levels, dB	*p* for interaction
Age, years		0.2301
<70	0.20 (0.07,0.33) 0.0034	
≥70	0.04(−0.19,0.26) 0.7344	
Gender		0.4198
Male	0.15(−0.00,0.30)0.0501	
Female	0.06(−0.10,0.22) 0.4488	
BMI		0.0565
<30	0.21 (0.08,0.35)0.0021	
≥30	−0.01(−0.19,0.18)0.9370	
Drink		0.4977
Yes	0.20(−0.00,0.40) 0.0512	
No	0.12(−0.01,0.25) 0.0689	
Diabetes		0.0726
Yes	−0.13(−0.43,0.17)0.3968	
No	0.16 (0.05.0.28) 0.0058	
Smoke		0.3797
Yes	0.16 (0.01.0.30) 0.0308	
No	0.06(−0.11.0.23) 0.4810	
Hypertension		0.8550
Yes	0.15 (−0.02, 0.31) 0.0786	
No	0.13 (−0.02, 0.27) 0.0852	
Noise exposure		0.7304
Yes	0.11 (−0.05, 0.27) 0.1661	
No	0.15 (−0.00, 0.30) 0.0563	

## 4 Discussion

In conclusion, our cross-sectional study of 8,535 participants showed a significant positive correlation between SII and changes in hearing thresholds, implying that SII, as an inflammatory marker, may become a biomarker of hearing loss in the future. Subgroup analyses and interaction tests indicated that this connection was constant across demographics. These results show an intense link between hearing loss and SII, offering essential data for further clinical and fundamental studies in this area.

Chronic inflammation is a significant factor in the onset of hearing loss, according to prior epidemiological studies. Recent research on animals has demonstrated that the cochlea’s local inflammation plays a role in the process of cell destruction and hearing loss (21, 22). Furthermore, numerous traditional inflammatory indicators have been employed extensively in clinical investigations, and plasma levels of inflammatory markers have been demonstrated to be increased in a variety of hearing loss types. For instance, a further cross-sectional investigation discovered that higher leukocyte counts may raise the chance of hearing loss (23). After examining the relationship between long-term CRP levels and the prevalence of hearing impairment in an aging population, Nash et al. came to the conclusion that long-term CRP levels predicted hearing impairment in those under the age of 60 at baseline (5). Intriguingly, the research by Shruti Gupta et al. found no statistically significant link between hearing loss and the plasma markers of inflammation CRP, IL-6, and TNFR-2 but did see greater TNFR-2 levels in women over 60 (4). Nonetheless, the absence of a clear association in these studies does not necessarily imply that there is no link between inflammatory markers and hearing loss. It is possible that inflammation occurring specifically at the cochlear level may not be accurately reflected in serum levels of markers such as CRP, IL-6, and TNFR-2. These markers may not be the most suitable for validating the relationship. The limitations of these markers can lead to a negative outcome in studies, which may obscure any potential association between inflammatory markers and hearing thresholds. Consequently, we turned to the SII metric, which has demonstrated excellent predictive capabilities as a diagnostic biomarker that may be responsive to systemic inflammatory activity. This prompted us to hypothesize whether systemic inflammation could serve as a biomarker for the risk of hearing loss. For instance, the SII was found to be a reliable predictive predictor in patients undergoing radical hepatectomy for ICC in the study by Zhang et al. Still, conventional inflammatory markers like neutrophil-to-lymphocyte ratio (NLR) had no bearing on the patient’s clinical outcomes (24).

In our study, we explore the association between the Systemic Immune-Inflammation Index (SII) and hearing loss. To further emphasize the strengths of our research, we refer to a series of studies, including analyses of the sensitivity and specificity of CRP, IIS, NLR, and PLR in hearing loss diseases and cohorts. The study by Verschuur et al. indicates that inflammatory markers, including CRP, are associated with hearing thresholds in older individuals (25). Shapira et al. explore the relationship between inflammatory markers such as NLR and PLR and hearing loss in young people (26). Their results further confirm the association between inflammatory markers and hearing loss across different age groups. Nash et al. highlight the potential value of CRP in predicting hearing loss by examining the relationship between long-term systemic inflammation and the cumulative incidence of age-related hearing loss (5). Ulu et al. further confirmed the importance of inflammatory markers in hearing loss by comparing the SII, NLR, and PLR values of patients with idiopathic sudden sensorineural hearing loss (9). This study provides important background for our work, suggesting that SII, as a comprehensive inflammatory index, may have a unique value in assessing the risk of hearing loss. Overall, by analyzing the sensitivity and specificity of CRP, IIS, NLR, and PLR in the same hearing loss disease and cohort, we can gain a more comprehensive understanding of the role of systemic inflammation in the development of hearing loss. These analyses not only highlight the strengths of our study but also provide new directions for future research to further explore the potential applications of inflammatory markers in the prevention and treatment of hearing loss. Similarly, SII has been found to be more effective than other inflammatory factors in similar studies of several different diseases (12, 27, 28). It is clear that SII, a novel marker of systemic inflammation, can provide additional clinical information in peripheral blood and lead to a more accurate diagnosis. In our cross-sectional study, the results showed a significant positive correlation between SII and changes in hearing thresholds for low, speech and high frequencies. More specifically, subjects in the highest SII tertile had a significantly increased risk of low frequency and speech hearing loss compared to the lowest SII tertile. At the same time, there was no significant association between tertile SII and high-frequency hearing loss. According to the traveling wave hypothesis, high-frequency sound waves vibrate the cochlea’s basal region, whereas low-frequency sound waves vibrate the cochlea’s apical portion. Therefore, the results of the current study imply that the SII may have a higher impact on the apical part of the cochlea, which calls for future research into the potential processes.

Inflammation has been linked to a number of neurodegenerative disorders, which include Alzheimer’s disease, Parkinson’s disease, and amyotrophic lateral sclerosis, in numerous studies. Many studies have shown that inflammation is closely related to various neurodegenerative diseases such as Alzheimer’s disease, Parkinson’s disease, and amyotrophic lateral sclerosis (ALS) (29). The neural pathways serve as a defense mechanism initially protecting the brain by eliminating or inhibiting multiple variables (30, 31). Factor reactions can have beneficial effects by promoting tissue repair and clearing cell debris. However, sustained factor responses are incremental and can inhibit regeneration. Researchers have found that compared to the non-progression of Alzheimer’s disease, TNF-α levels, a pro-inflammatory cytokine, have increased in the cerebrospinal fluid of patients with cognitive impairment due to Alzheimer’s disease, while TNF-β, an anti-inflammatory cytokine, has shown relatively low levels (32). Scholars have proposed that neurological disorders are a common pathological mechanism in ALS patients with or without genetic mutations, characterized by the respiration of activated small glial cells and astrocytes. Activated small glial cells and astrocytes producing pro-inflammatory cytokines are upregulated in postmortem tissues of ALS patients (33, 34). This indicates that inflammation plays a crucial role in the process of neurodegenerative injury (35–37). Still, there is just a tiny amount of research examining the possible link between systemic inflammation and hearing loss. The underlying mechanisms of age-related hearing loss and cognitive frailty may be accelerated by systemic inflammation, according to a recent study that found a relationship between age-related abnormalities in central auditory processing and mental deficiency (38). The acoustic center and hippocampus are affected, which increases the risk of hearing loss and cognitive decline. Persistent inflammation has the potential to alter brain neuroplasticity and stimulate glial cells, causing a long-lasting inflammatory state that further damages neurons and causes them to degenerate (39, 40).

Contrary to one idea, hearing loss is caused by oxidative stress and a hyperactive neuroinflammatory response, and oxidative stress and mitochondrial malfunction have the potential to speed up apoptosis, affect cochlear degeneration, and velocity up aging (41, 42). Likewise, because systemic homeostasis is maintained via the inner ear’s vasculature, inflammation is not restricted to the inner ear. Exposing vital cochlear veins to circulating inflammatory substances throughout the body compromises the vascular barrier and increases the risk of hearing loss (6, 43).

The current investigation possesses many types of substantial advantages. The credibility of our findings is firstly increased by the use of a sizable, nationally representative sample and the adjustment for a variety of factors. The widely accepted gold standard PTA, which measures hearing loss, further supports the validity of our findings. Nevertheless, some limitations cannot be avoided. Due to the inherent shortcomings of cross-sectional studies, we were not able to conduct a causal assessment. Therefore, prospective studies with larger sample sizes are needed to clarify causality. Furthermore, there are subgroups of hearing loss based on etiology (noise hearing loss, conductive hearing loss, age-related hearing loss, etc.). However, since the NHANES does not include these particular data, more research is required to address this limitation and determine whether the SII is applicable in a wider context. Therefore, further research is necessary to understand the pathogenic mechanisms underlying hearing loss more thoroughly. Such research should include studies using animal models and prospective examinations.

## 5 Conclusion

Together, these studies provide evidence of a link between SII and hearing loss and are crucial for expanding our knowledge of the relationship between systemic inflammation and hearing loss. However, to confirm our findings and create a more complete understanding of this link, deeper research and rigorous prospective investigations are essential.

## Data availability statement

Publicly available datasets were analyzed in this study. This data can be found here: Centers for Disease Control and Prevention (CDC), National Centers for Health Statistics (NCHS), and National Health and Nutrition Examination Survey (NHANES) database, https://wwwn.cdc.gov/nchs/nhanes/Default.aspx, NHANES 2009–2018.

## Ethics statement

Ethical review and approval was not required for the study on human participants in accordance with the local legislation and institutional requirements. Written informed consent from the patients/participants or patients/participants’ legal guardian/next of kin was not required to participate in this study in accordance with the national legislation and the institutional requirements.

## Author contributions

TZ: Conceptualization, Formal analysis, Methodology, Visualization, Writing – original draft. JM: Data curation, Conceptualization, Validation, Visualization, Software, Writing – review & editing. PZ: Methodology, Formal analysis, Visualization, Writing – review & editing. XYu: Formal analysis, Software, Visualization, Writing – review & editing. XYa: Project administration, Supervision, Writing – review & editing.
