# Supplementary material for: Association between the systemic immuno-inflammation index and hearing loss: result from NHANES 2009–2018
Source: Front Neurol. 2024 Apr 23;15:1369492. doi: 10.3389/fneur.2024.1369492 (PMC11074419; doi:10.3389/fneur.2024.1369492)
Supplement: Supplementary file 1 [file Table_1.DOCX]

**Supplemental Table S1**

| OBS components | Assignment scheme | | |
| --- | --- | --- | --- |
|  | 0 | 1 | 2 |
| Lifestyle prooxidants |  |  |  |
| Alcohol (g/d) | ≥30 for male  ≥15 for female | 0-30 for male 0-15 for female | None |
| Body mass index (kg/m2) | >30 | 25-30 | <25 |
| Cotinine (ng/mL) | >0.038 | 0.038-1.13 | <1.13 |
| Lifestyle antioxidants |  |  |  |
| Physical activity (MET-minute/week) | <400 | 400-1,000 | >1,000 |
| Dietary prooxidants |  |  |  |
| Total fat (g/d) | >107.43 | 69.83-107.43 | <69.83 |
| Iron (mg/d) | >19.17 | 12.88-19.17 | <12.88 |
| Dietary antioxidants |  |  |  |
| Dietary fiber (g/d) | <12.56 | 12.56-19.70 | >19.70 |
| Carotene (RE/d) | <98.83 | 98.83-306.25 | >306.25 |
| Riboflavin (mg/d) | <1.79 | 1.79-2.69 | >2.69 |
| Niacin (mg/d) | <20.65 | 20.65-29.75 | >29.75 |
| Total folate (mcg/d) | <316.00 | 316.00-492.00 | >492.00 |
| Vitamin B6 (mg/d) | <1.59 | 1.59-2.40 | >2.40 |
| Vitamin B12 (mcg/d) | <3.36 | 3.36-6.20 | >6.20 |
| Vitamin C (mg/d) | <42.44 | 42.44-113.21 | >113.21 |
| Vitamin E (ATE) (mg/d) | <5.82 | 5.82-9.42 | >9.42 |
| Calcium (mg/d) | <646.00 | 646.00-1072.00 | >1072 |
| Magnesium (mg/d) | <257.00 | 257.00-361.28 | >361.28 |
| Zinc (mg/d) | <9.75 | 9.75-15.10 | >15.10 |
| Copper (mg/d) | <1.12 | 1.12-1.57 | >1.57 |
| Selenium (mcg/d) | <94.94 | 94.94-141.80 | >141.80 |

Notes：Assignment scheme of Oxidative Balance Score.
OBS, oxidative balance score; RE, retinol equivalent; ATE, alpha-tocopherol; MET, metabolic equivalent.
